# Magnesium sulfate treatment for juvenile ferrets following induction of hydrocephalus with kaolin

**DOI:** 10.1186/2045-8118-12-S1-P28

**Published:** 2015-09-18

**Authors:** Domenico Luciano Di Curzio, Emily Turner-Brannen, Xiaoyan Mao, Marc Del Bigio

**Affiliations:** 1 University of Manitoba, Canada; 2 Children's Hospital Research Institute of Manitoba, Canada

## Introduction

Hydrocephalus is characterized by altered cerebrospinal fluid flow increasing brain ventricular cavities. Rodent studies showed that axonal pathology includes calcium-mediated proteolysis, which can be reduced by the calcium channel antagonist magnesium sulfate (MgSO4). Hydrocephalic ferrets show similar neurological changes as rodents and humans, and thus MgSO4 treatment was tested in juvenile ferrets.

## Methods

Fourteen-day old ferrets were given kaolin injections into the cisterna magna. Magnetic resonance imaging was performed two weeks later to assess ventricle size and stratify ferrets into treatment groups. Ferrets were treated for two weeks with MgSO4 or saline, and then imaged before sacrifice. Behaviour was examined thrice weekly. Histological and biochemical assays were also performed.

## Results

Compared to controls, hydrocephalic ferrets were not appreciably different in terms of weight and behaviour; however, those receiving MgSO4 weighed less, were more lethargic, and displayed reduced activity than those receiving saline. Hydrocephalic ferrets developed ventriculomegaly, but there were no differences for either treatment group. They also exhibited cerebral thinning, decreased depth of cerebral sulci, and rarefaction and fragmentation of periventricular white matter. Though glial fibrillary acidic protein content was elevated in saline treated ferrets, indicative of reactive astroglial changes, there were no significant differences compared to MgSO4 treated ferrets nor to controls. Myelin basic protein content and myelin enzyme activity also displayed no significance differences between treatment groups.

## Conclusions

Hydrocephalus-induced disturbances are not ameliorated by MgSO4 treatment. This suggests that unlike rodents, hydrocephalic ferrets do not experience behavioural improvements nor white matter protection from MgSO4 therapy, which may be the case for humans with even more complex brains.
